# Independent and Interactive Effects of Habitually Ingesting Fermented Milk Products Containing *Lactobacillus casei* Strain Shirota and of Engaging in Moderate Habitual Daily Physical Activity on the Intestinal Health of Older People

**DOI:** 10.3389/fmicb.2019.01477

**Published:** 2019-07-31

**Authors:** Yukitoshi Aoyagi, Ryuta Amamoto, Sungjin Park, Yusuke Honda, Kazuhito Shimamoto, Akira Kushiro, Hirokazu Tsuji, Hoshitaka Matsumoto, Kensuke Shimizu, Kouji Miyazaki, Satoshi Matsubara, Roy J. Shephard

**Affiliations:** ^1^Exercise Sciences Research Group, Tokyo Metropolitan Institute of Gerontology, Tokyo, Japan; ^2^Food Research Department, Yakult Central Institute, Tokyo, Japan; ^3^Microbiological Research Department, Yakult Central Institute, Tokyo, Japan; ^4^Basic Research Department, Yakult Central Institute, Tokyo, Japan; ^5^Faculty of Kinesiology and Physical Education, University of Toronto, Toronto, ON, Canada

**Keywords:** aging, constipation, defecation, exercise intensity, gut microbiota, intestinal motility, probiotics, step count

## Abstract

Infrequent bowel movements decrease the number of beneficial bacteria in the human intestines, thereby potentially increasing the individual’s risk of colorectal cancer. The correction of such bowel problems could therefore make an important contribution to improving population health and quality-adjusted lifespan. We examined independent and interactive effects upon the fecal microbiota of two potentially favorable determinants of intestinal motility: the intake frequency of a fermented milk product containing *Lactobacillus casei* strain Shirota (LcS) and the quantity/quality of habitual physical activity in 338 community-living Japanese aged 65–92 years. Subjects were arbitrarily grouped on the basis of questionnaire estimates of LcS intake (0–2, 3–5, and 6–7 days/week) and pedometer/accelerometer-determined patterns of physical activity [<7000 and ≥7000 steps/day, or <15 and ≥15 min/day of activity at an intensity >3 metabolic equivalents (METs)]. After adjustment for potential confounders, the respective numbers of various beneficial fecal bacteria tended to be larger in more frequent consumers of LcS-containing products, this trend being statistically significant (mostly *P* < 0.001) for total *Lactobacillus*, the *Lactobacillus casei* subgroup, and the *Atopobium* cluster; in contrast, there were no statistically significant differences in fecal bacterial counts between the physical activity groups. A multivariate-adjusted logistic regression analysis estimated that the risk of infrequent bowel movements (arbitrarily defined as defecating ≤3 days/week) was significantly lower (*P* < 0.05) in subjects who ingested LcS-containing products 6–7 rather than 0–2 days/week [odds ratio (95% confidence interval) 0.382 (0.149–0.974)] and was also lower in those who took ≥7000 rather than <7000 steps/day [0.441 (0.201–0.971)] or spent ≥15 rather than <15 min/day of physical activity at an intensity >3 METs [0.412 (0.183–0.929)]. The risk of infrequent bowel movements in subjects who combined 6–7 days/week of LcS with ≥7000 steps/day or ≥15 min/day of activity at >3 METs was only a tenth of that for individuals who combined 0–2 days/week of LcS with <7000 steps/day or <15 min/day at >3 METs. These results suggest that elderly individuals can usefully ingest LcS-containing supplements regularly (≥6 days/week) and also engage in moderate habitual physical activity (≥7000 steps/day and/or ≥15 min/day at >3 METs) in order to enhance their gastrointestinal health.

## Introduction

There are many different factors that affect human gastrointestinal health, including a healthy diet with a high fiber content. Certain types/species of microbiota are also important to gastrointestinal health, and the processes involved appear to be facilitated by the regular ingestion of fermented milk products containing lactic acid bacteria. A specific bacterium, the *Lactobacillus casei* strain Shirota (LcS) has been used in the production of fermented milk for more than 80 years, and products containing LcS have become very popular both in Japan and world-wide; respective consumptions averaged about 8 and 37 million bottles per day in the year 2016 (Yakult Honsha Co. Ltd., 2017).

Clinical trials have shown that LcS has many beneficial effects, including protection against infection (Gleeson et al., 2011; Nagata et al., 2011; Sur et al., 2011; Shida et al., 2017), enhanced immunoregulation (Nagao et al., 2000; Tamura et al., 2007), a reduced risk of carcinogenesis (Aso et al., 1995; Ohashi et al., 2002; Ishikawa et al., 2005; Toi et al., 2013), and the control of blood pressure (Nakajima et al., 1995; Aoyagi et al., 2017). Moreover, ingestion of LcS enhances intestinal motility, facilitating defecation in young and middle-aged individuals with either hard or soft stools (Matsumoto et al., 2006, 2010; Shioiri et al., 2006; Sakai et al., 2011). However, the impact of regular LcS ingestion on the risk of sustained problems of bowel movement in ostensibly healthy community-dwelling older people merits further epidemiological study.

Infrequent bowel movements reduce a person’s quality of life, and can have serious medical and social impacts (Johanson and Kralstein, 2007). The cumulative incidence of chronic constipation is higher in the elderly than in younger people (Choung et al., 2007). In 2016, 7.4% of Japanese elderly people (6.5% for men and 8.1% for women) complained of constipation (Ministry of Health, Labour and Welfare, Japan, 2016a). One immediate concomitant of infrequent bowel movements is a decrease of beneficial bacteria such as *Bifidobacterium*, *Lactobacillus*, and *Roseburia* species in the intestinal microbiota (Khalif et al., 2005; Kim et al., 2015; Mancabelli et al., 2017), with a resultant increase in the intestinal production of toxic and mutagenic compounds such as phenol and *p*-cresol, and a decrease in concentrations of butyric acid, a known anticancer substance (Ara et al., 2002; Nakabayashi et al., 2011; Kano et al., 2013). These changes could potentially increase the risk of colorectal cancer (Kotake et al., 1995; Le Marchand et al., 1997; Jacobs and White, 1998; Roberts et al., 2003), which has now become the leading cause of death among the Japanese (Ministry of Health, Labour and Welfare, Japan, 2016b).

Studies in the prevention and control of constipation (Sandler et al., 1990; Dukas et al., 2003; De Schryver et al., 2005) have to date suggested an association between inadequate habitual physical activity and infrequent bowel movements, although most investigations of this topic have been based simply upon questionnaire reports of exercise frequency per week. In consequence, there remains a need to define relationships between accurate objective measurements of habitual physical activity, defecation patterns and intestinal microbiota, particularly in the elderly, where both low levels of physical activity and constipation are frequent issues. Over an 18-year period since 2000, we have been conducting an epidemiological study in the community of Nakanojo Town, Gunma Prefecture, Japan (the Nakanojo Study), monitoring patterns of habitual physical activity in people aged 65 years and older (Aoyagi and Shephard, 2009, 2010, 2011, 2013). Accurate objective measurements of physical activity have been made 24 h per day, 365 days a year, using a physical activity monitor with a built-in accelerometer, and these data provide an exciting opportunity to explore associations between habitual activity and intestinal health.

The purpose of the present study was thus to examine associations between the intake frequency of fermented milk products containing LcS, the quantity and quality of habitual physical activity, and the intestinal health of free-living older people.

## Materials and Methods

### Subjects

The subjects were 140 male and 198 female self-supporting and independent Japanese volunteers aged 65–92 years who had been recruited to the Nakanojo Study (Aoyagi and Shephard, 2009, 2010, 2011, 2013). Subjects gave their written informed consent to participation in a study approved by the ethics review committee of the Tokyo Metropolitan Institute of Gerontology, after the protocol, stresses, and possible risks had been fully explained to them. Criteria of recruitment included attendance at an annual medical examination, functional independence, and the absence of chronic or progressive conditions that could limit physical activity or have a major effect on the individual’s perceived quality of life (e.g., cancer, arthritic diseases, Parkinson’s disease, Alzheimer’s disease, multiple sclerosis, amyotrophic lateral sclerosis, and dementia).

### Estimation of the Frequency of Ingestion of Fermented Milk Products

The frequency of ingestion of fermented milk products containing LcS was estimated by a self-administered questionnaire with pictures of a series of commercially available LcS-containing products, including “Yakult”, “Joie”, “Soful”, and “Pretio” (Yakult Honsha Co. Ltd., Minato, Tokyo, Japan), each of which contains 0.9–40 billion live LcS per bottle. Subjects were asked how many days per week they had ingested products of the type illustrated over a 1-month period before the collection of fecal samples. The frequency of intake of general fermented milk products, such as yogurt (including the above Yakult Honsha Co. Ltd. products), was estimated in the same manner (see Supplementary Table 1). Subjects were classed as consuming a bottle of the product 0–2 days/week (designated as “group 0–2”), 3–5 days/week (designated as “group 3–5”), or 6–7 days/week (designated as “group 6–7”).

### Measurement of Physical Activity Patterns

Physical activity patterns were measured for 24 h per day over a 1-month period, using a uniaxial acceleration sensor (Lifecorder; Suzuken Co. Ltd., Nagoya, Aichi, Japan), as described previously (Aoyagi and Shephard 2009, 2010, 2011, 2013). The average number of steps taken per day and the daily cumulative duration of moderate-intensity exercise [activity demanding an energy expenditure greater than 3 metabolic equivalents (METs)] were calculated for each subject (see Supplementary Table 2). Subjects were classed as taking <7000 steps/day (designated as “group <7000”) or ≥7000 steps/day (designated as “group ≥7000”) and spending <15 min/day at >3 METs (designated as “group <15”) or ≥15 min/day at >3 METs (designated as “group ≥15”). As previously demonstrated (Aoyagi and Shephard, 2009, 2010, 2011, 2013), a series of cross-sectional and/or longitudinal analyses has shown a low prevalence and/or incidence of various chronic diseases and disorders (including cancer of the colon and rectum; Aoyagi and Shephard, 2011) in elderly individuals taking daily doses of physical activity above disease-specific thresholds.

### Assessment of Anthropometric Characteristics and Blood Profiles

The physical characteristics of subjects (age, sex, height, body mass, body mass index, abdominal circumference, body fat percentage, fat mass, fat-free mass, and muscle mass) were determined by standard anthropometric techniques (Shephard et al., 2013). Biochemical profiles (triglyceride, high-density lipoprotein cholesterol, low-density lipoprotein cholesterol, glycosylated hemoglobin A_1c_, blood sugar, glutamic oxaloacetic transaminase, glutamic pyruvic transaminase, γ-glutamyl transpeptidase, albumin, creatinine, uric acid concentrations, and the estimated glomerular filtration rate) were also measured by standard methods (Health Sciences Research Institute Inc., Yokohama, Kanagawa, Japan).

### Examination of Physical Health

Blood pressure was measured after 5 min of seated rest, using an automatic sphygmomanometer (BP-103iII; Colin Medical Technology Co. Ltd., Komaki, Aichi, Japan). At least one further measurement was made after a further 5 min of rest if the initial reading suggested that an individual had become hypertensive (or rarely, hypotensive). Preferred and maximal walking speeds were determined over a 5-m distance, using a stopwatch (SVAE101; Seiko Corp., Minato, Tokyo, Japan), as described previously (Aoyagi et al., 2004). Subjects completed two trials to determine each of comfortable and maximal walking speeds; the averaged and the higher velocities were each recorded for each of the two measurements. Peak handgrip force was assessed for the dominant hand, using a Smedley dynamometer (ES-100; Evernew Co. Ltd., Koto, Tokyo, Japan). Two trials were performed, and the larger of the two readings was noted. Quantitative ultrasound measurements of osteosonic index for the calcaneus were made using an Achilles ultrasonic bone densitometer (AOS-100; Aloka Co. Ltd., Mitaka, Tokyo, Japan), as described previously (Shephard et al., 2017).

### Investigation of Nutrient Intake

The nutritional status of the subjects was evaluated by a certified nutritionist over a 1-week period, using Version 3.5 of the Food Frequency Questionnaire Based on Food Groups (FFQg; Kenpakusha Co. Ltd., Bunkyo, Tokyo, Japan), which is a 20-item questionnaire regarding the consumption of items from 29 food groups and 10 methods of food preparation. On the basis of responses to this questionnaire, the daily intake of energy, nutrients, and food groups was estimated for the 1- to 2-month period prior to the start of the study. The estimated nutrients included protein, lipid, carbohydrate, dietary fiber, saturated fatty acids, monounsaturated fatty acids, polyunsaturated fatty acids, cholesterol, sodium, potassium, calcium, magnesium, iron, and vitamin C.

### Collection and Treatment of Fecal Samples

Subjects collected freshly excreted stool samples of around 500 mg into sterile feces tubes (Sarstedt AG & Co., Nümbrecht, Nordrhein-Westfalen, Germany) containing 2 ml of a ribonucleic acid (RNA) stabilization solution (RNA*later*; Ambion Inc., Austin, TX, United States). Samples were kept at room temperature and sent to the Yakult Central Institute within a couple of days. After arrival at the Institute, they were stored at 4°C in a biosafety level 2 laboratory until processing. Fecal samples were weighed, suspended in nine volumes of RNA*later*, and then homogenized. The fecal homogenate (200 μl) was added to 1 ml of sterilized phosphate-buffered saline and then centrifuged at 10,000 × *g* for 5 min. The supernatant was discarded, and the pellet was stored at −80°C until RNA and deoxyribonucleic acid (DNA) extraction.

### Assay of Gut Microbiota

The number of each of the many bacteria detected in the gut was determined by the reverse transcription-quantitative polymerase chain reaction (RT-qPCR) technique (Matsuda et al., 2007, 2009, 2012; Kubota et al., 2010; Tsuji et al., 2012; Nagpal et al., 2016; Suzuki et al., 2017), using the Yakult Intestinal Flora Scan (YIF-SCAN; Yakult Honsha Co. Ltd., Minato, Tokyo, Japan). In the preliminary/preparatory stages of this determination, RNA was isolated from fecal samples, as described previously (Matsuda et al., 2012), and it was used for RT-qPCR. The specificity and sensitivity of RT-qPCR analysis for selected gut bacteria using group-, genus-, species-, and strain-specific primer sets have been described previously (Matsuda et al., 2007, 2009, 2012). Base sequences of the primer sets, annealing temperatures, and minimum detection limits for quantification of the bacteria are shown in Supplementary Table 3. A detection limit was assigned for the statistical calculation of fecal bacterial counts. Two beneficial bacteria (*Lactobacillus* and *Bifidobacterium*), four pathogenic bacteria (*Pseudomonas*, *Staphylococcus*, *Clostridium difficile*, and *Clostridium perfringens*), five obligate anaerobes (*Atopobium* cluster, *Bacteroides fragilis* group, *Clostridium coccoides* group, *Clostridium leptum* subgroup, and *Prevotella*), and three facultative anaerobes (Enterobacteriaceae, *Enterococcus*, and *Streptococcus*) were selected because of their predominance in the intestines of healthy Japanese people (Matsuda et al., 2009; Tsuji et al., 2012, 2018). Total *Lactobacillus* (*L.*) was calculated as the sum of the counts of six subgroups (*L. casei*, *L. gasseri*, *L. plantarum*, *L. reuteri*, *L. ruminis*, and *L. sakei*) and two species (*L. fermentum* and *L. brevis*). On the other hand, the relative abundances of the fecal bacteria families were determined by amplicon analysis targeting the 16S ribosomal RNA (rRNA) gene, as described previously (Kato-Kataoka et al., 2016), using the Quantitative Insights Into Microbial Ecology (QIIME) software (Caporaso et al., 2010). In brief, DNA was isolated from fecal samples (Fujimoto et al., 2008), and the V1-V2 region of the 16S rRNA gene of gut microbiota was amplified using the forward 27Fmod2-MiSeqV2 and the reverse 338RMiSeqV2-001 primers (see Supplementary Table 4 for details) to monitor DNA amplification in a real-time PCR (Applied Biosystems 7500; Life Technologies Japan Ltd., Minato, Tokyo, Japan). The amplified DNA was purified using a PCR product purification kit (Agencourt AMPure XP; Beckman Coulter K. K., Koto, Tokyo, Japan), quantified using a double-stranded DNA (dsDNA) assay kit (Quant-iT PicoGreen dsDNA; Life Technologies Japan Ltd., Minato, Tokyo, Japan), and sequenced using a MiSeq sequencing system (Illumina K. K., Minato, Tokyo, Japan).

### Evaluation of Defecation

The frequency of defecation was estimated by a self-administered questionnaire. Subjects were asked how many days they had defecated over a 1-week period before the fecal sampling. Infrequent bowel movements were arbitrarily defined as defecating 3 days or less per week. The consistency of the stools was estimated using the Bristol Stool Form Scale (BSFS; Heaton et al., 1992), a diagnostic tool designed to classify the shape and type of human feces into seven distinct categories: separate hard lumps, like nuts (=1); sausage-shaped, but lumpy (=2); like a sausage, but with cracks on its surface (=3); like a sausage or snake, smooth and soft (=4); soft blobs with clear-cut edges (=5); fluffy pieces with ragged edges, a mushy stool (=6); and watery, no solid pieces, entirely liquid (=7).

### Statistical Analyses

Version 23.0 of the IBM SPSS Statistics (IBM Corp., Armonk, NY, United States) was used throughout. Subjects were divided into arbitrary groups, based on the reported frequency of ingestion of fermented milk products (0–2, 3–5, and 6–7 days/week) and the pattern of habitual physical activity (<7000 and ≥7000 steps/day, or <15 and ≥15 min/day at >3 METs). Analyses of covariance assessed independent differences among or between groups with respect to anthropometry, supplement consumption, physical activity, physical health, nutrition, blood, fecal microbiota, and fecal characteristics, after controlling data for age, sex, body mass index, smoking status, and/or alcohol consumption. Chi-square tests assessed differences in male/female ratio and the rate of infrequent bowel movements among and/or between groups. Logistic regression analyses determined odds ratios and 95% confidence intervals adjusted for age, sex, body mass index, smoking status and alcohol consumption, and assessed independent relationships between fermented milk product intakes and/or daily physical activity patterns and the estimated risk of infrequent bowel movements. All statistical contrasts were made at the 0.05 level of significance, although the appropriate Tukey correction was applied when multiple comparisons were made.

## Results

Two hundred and four, 54, and 80 of the 338 subjects consumed fermented milk products containing LcS (as manufactured by the Yakult Honsha Company) 0–2, 3–5, and 6–7 days/week, respectively; with respect to overall fermented milk products, the corresponding numbers were 78, 70, and 190 of the 338 participants (Supplementary Table 1). In terms of habitual physical activity, 187 and 151 of the 338 subjects took <7000 and ≥7000 steps/day, respectively, and 188 and 150 of subjects spent <15 and ≥15 min/day of physical activity at an intensity >3 METs, respectively (Supplementary Table 2). Associations with nutrition and intestinal health, particularly the frequency of defecation, were similar for the various methods of classifying our sample, and we thus limit the discussion of findings mainly in relation to the ingestion of LcS-containing fermented milk products and the duration of physical activity >3 METs.

Initially, there were no statistically significant differences among 0–2, 3–5, and 6–7 LcS consumption groups with respect to any of the variables examined, except age and the intake of a few nutrients (potassium, calcium, and iron). The most frequent users of LcS were older and tended to be better nourished than their peers (Table 1). However, statistically significant differences were found between <15 and ≥15 physical activity groups in terms of age, body mass, abdominal circumference, body fat percentage, fat mass, preferred and maximal walking speeds; physically more active subjects were younger, less fat, and faster walkers (Table 2). Current smokers were 16 of 204 in the LcS group 0–2, 2 of 54 in the LcS group 3–5, 3 of 80 in the LcS group 6–7, 11 of 188 in the activity group <15, and 10 of 150 in the activity group ≥15. Moderate alcohol consumption was reported by 83 of 204 subjects in group 0–2, 21 of 54 subjects in group 3–5, 22 of 80 subjects in group 6–7, 62 of 188 subjects in group <15, and 64 of 150 subjects in group ≥15, with the remaining subjects not consuming significant amounts of alcohol.

**TABLE 1:** Characteristics of subjects consuming fermented milk products containing *Lactobacillus casei* strain Shirota (LcS) 0–2, 3–5, or 6–7 days per week.

	**0–2 days/week**		**3–5 days/week**		**6–7 days/week**	
			
	***n***	**Mean ± SD**	***n***	**Mean ± SD**	***n***	**Mean ± SD**
**Anthropometric parameters**						
Age (years)	204	73.6 ± 6.0	54	74.6 ± 5.4	80	76.3 ± 6.3^††^
Sex (male/female, %)	204	43.1 / 56.9	54	29.6 / 70.4	80	45.0 / 55.0
Height (m)	204	1.56 ± 0.09	54	1.54 ± 0.07	80	1.54 ± 0.09
Body mass (kg)	204	56.2 ± 9.7	54	54.5 ± 10.5	80	54.6 ± 9.3
Body mass index (kg/m^2^)	204	22.9 ± 2.9	54	23.0 ± 3.7	80	22.8 ± 2.9
Abdominal circumference (m)	172	0.84 ± 0.09	46	0.83 ± 0.10	73	0.83 ± 0.10
Body fat percentage (%)	192	25.4 ± 7.9	51	27.8 ± 8.1	78	24.5 ± 9.1
Fat mass (kg)	192	14.4 ± 5.5	51	15.3 ± 6.4	78	13.4 ± 5.6
Fat-free mass (kg)	192	41.7 ± 7.8	51	38.7 ± 7.2	78	40.8 ± 8.0
Muscle mass (kg)	192	39.5 ± 7.5	51	36.6 ± 6.8	78	38.6 ± 7.6
**Physical health measurements**						
Systolic blood pressure (mmHg)	198	129 ± 17	51	131 ± 19	79	130 ± 18
Diastolic blood pressure (mmHg)	198	76 ± 9	51	76 ± 11	79	75 ± 10
Preferred walking speed (m/s)	183	1.39 ± 0.21	48	1.40 ± 0.22	76	1.32 ± 0.21
Maximal walking speed (m/s)	183	2.12 ± 0.36	48	1.98 ± 0.36	76	2.05 ± 0.42
Peak handgrip force (N)	185	278 ± 86	48	248 ± 73	76	270 ± 80
Calcaneal osteosonic index (×10^6^)	193	2.38 ± 0.31	51	2.31 ± 0.33	79	2.33 ± 0.29
**Fermented milk product consumptions**						
LcS fermented milk products (days/week)	204	0.4 ± 0.7	54	4.1 ± 0.9^†⁣††^	80	6.8 ± 0.4^†⁣††,‡⁣‡‡^
Overall fermented milk products (days/week)	204	4.0 ± 2.9	54	5.0 ± 1.4^††^	80	6.9 ± 0.3^†⁣††,‡⁣‡‡^
**Habitual physical activity patterns**						
Step count (steps/day)	204	7185 ± 3189	54	6526 ± 2516	80	7134 ± 3684
Duration of exercise >3 METs (min/day)	204	18.1 ± 15.1	54	15.0 ± 13.9	80	16.8 ± 16.5
**Nutrient intakes**						
Energy (kcal/day)	176	2081 ± 581	47	2133 ± 501	67	2213 ± 698
Protein (g/day)	176	75.7 ± 23.8	47	78.9 ± 18.6	67	83.3 ± 30.4
Lipid (g/day)	176	68.4 ± 25.4	47	71.5 ± 22.2	67	77.4 ± 34.2
Carbohydrate (g/day)	176	278 ± 75	47	280 ± 66	67	287 ± 92
Dietary fiber (g/day)	176	17.1 ± 5.6	47	17.1 ± 5.2	67	18.5 ± 6.5
Saturated fatty acid (g/day)	176	21.1 ± 8.5	47	21.2 ± 6.6	67	24.1 ± 10.6
Monounsaturated fatty acid (g/day)	176	22.8 ± 8.7	47	23.5 ± 7.7	67	25.4 ± 13.1
Polyunsaturated fatty acid (g/day)	176	14.5 ± 5.4	47	15.4 ± 4.8	67	15.9 ± 6.9
Cholesterol (mg/day)	176	335 ± 122	47	347 ± 106	67	362 ± 149
Sodium (mg/day)	176	4752 ± 1857	47	4792 ± 1832	67	4918 ± 1701
Potassium (mg/day)	176	2870 ± 998	47	2835 ± 848	67	3223 ± 1097^†^
Calcium (mg/day)	176	753 ± 280	47	775 ± 214	67	875 ± 276^††^
Magnesium (mg/day)	176	297 ± 97	47	306 ± 82	67	330 ± 109
Iron (mg/day)	176	9.2 ± 3.4	47	9.8 ± 3.5	67	10.5 ± 3.8^†^
Vitamin C (mg/day)	176	125 ± 60	47	119 ± 55	67	140 ± 59
**Blood profiles**						
Triglyceride (mmol/l)	155	1.58 ± 1.19	40	1.39 ± 0.69	68	1.51 ± 0.87
High-density lipoprotein cholesterol (mmol/l)	155	1.58 ± 0.43	40	1.61 ± 0.48	68	1.59 ± 0.46
Low-density lipoprotein cholesterol (mmol/l)	155	3.15 ± 0.75	40	3.18 ± 0.80	68	3.15 ± 0.66
Glycosylated hemoglobin A_1c_ (%)	155	5.72 ± 0.55	40	5.63 ± 0.37	68	5.72 ± 0.53
Blood sugar (mmol/l)	155	6.16 ± 1.52	40	6.02 ± 1.07	68	6.38 ± 1.92
Glutamic oxaloacetic transaminase (IU/l)	155	25.0 ± 7.0	40	25.6 ± 6.2	68	24.4 ± 6.0
Glutamic pyruvic transaminase (IU/l)	155	20.1 ± 8.1	40	19.6 ± 6.4	68	19.7 ± 8.4
γ-glutamyl transpeptidase (IU/l)	155	27.9 ± 27.2	40	22.8 ± 13.3	68	21.5 ± 8.5
Albumin (g/l)	155	44.0 ± 2.5	40	43.3 ± 2.5	68	43.5 ± 2.8
Creatinine (μmol/l)	155	69.3 ± 15.5	40	66.9 ± 17.4	68	68.7 ± 15.1
Uric acid (μmol/l)	155	304 ± 76	40	311 ± 76	68	280 ± 59
Estimated glomerular filtration rate (ml/min/1.73 m^2^)	155	65.4 ± 12.2	40	65.2 ± 14.7	68	65.4 ± 11.8

*METs, metabolic equivalents; SD, standard deviation. Intergroup differences in male/female ratio were assessed by chi-square tests. Linear trends for three-group differences in each of the other variables were assessed by analyses of covariance, after adjusting data on age for sex and the others for age and sex; if the trends were statistically significant, *post hoc* Tukey’s tests assessed two-group differences in each variable. ^†^*P* < 0.05, ^†^^†^*P* < 0.01, and ^†^^†^^†^*P* < 0.001, respectively, versus 0–2 days/week; ^‡^^‡^^‡^*P* < 0.001 versus 3–5 days/week. Data on overall fermented milk products are not shown, mainly because of their similarities to those on LcS-containing products.*

**TABLE 2:** Characteristics of subjects taking exercise demanding an energy expenditure >3 metabolic equivalents (METs) for <15 or ≥15 min per day.

	**<15 min/day at >3 METs**		**≥15 min/day at >3 METs**	
		
	***n***	**Mean ± SD**	***n***	**Mean ± SD**
**Anthropometric parameters**				
Age (years)	188	75.8 ± 6.2	150	72.7 ± 5.4^*⁣**^
Sex (male/female, %)	188	41.0 / 59.0	150	42.0 / 58.0
Height (m)	188	1.55 ± 0.08	150	1.56 ± 0.09
Body mass (kg)	188	56.0 ± 9.6	150	55.1 ± 10.0^*^
Body mass index (kg/m^2^)	188	23.2 ± 3.3	150	22.6 ± 2.7
Abdominal circumference (m)	168	0.85 ± 0.09	123	0.82 ± 0.09^**^
Body fat percentage (%)	178	26.8 ± 8.3	143	24.1 ± 8.0^**^
Fat mass (kg)	178	15.1 ± 5.9	143	13.3 ± 5.3^**^
Fat-free mass (kg)	178	40.6 ± 7.5	143	41.4 ± 8.2
Muscle mass (kg)	178	38.5 ± 7.1	143	39.2 ± 7.8
**Physical health measurements**				
Systolic blood pressure (mmHg)	184	131 ± 18	144	127 ± 17
Diastolic blood pressure (mmHg)	184	76 ± 10	144	75 ± 9
Preferred walking speed (m/s)	167	1.30 ± 0.20	140	1.47 ± 0.20^*⁣**^
Maximal walking speed (m/s)	167	2.00 ± 0.40	140	2.17 ± 0.34^**^
Peak handgrip force (N)	169	264 ± 84	140	281 ± 82
Calcaneal osteosonic index (×10^6^)	179	2.32 ± 0.32	144	2.40 ± 0.30
**Fermented milk product consumptions**				
LcS fermented milk products (days/week)	188	2.6 ± 2.8	150	2.4 ± 2.9
Overall fermented milk products (days/week)	188	4.8 ± 2.6	150	4.9 ± 2.6
**Habitual physical activity patterns**				
Step count (steps/day)	188	5116 ± 1758	150	9514 ± 2953^*⁣**^
Duration of exercise >3 METs (min/day)	188	7.3 ± 4.1	150	29.9 ± 14.8^*⁣**^
**Nutrient intakes**				
Energy (kcal/day)	157	2085 ± 557	133	2162 ± 644
Protein (g/day)	157	76.4 ± 25.1	133	79.8 ± 24.6
Lipid (g/day)	157	70.1 ± 27.8	133	72.1 ± 26.9
Carbohydrate (g/day)	157	276 ± 76	133	286 ± 80
Dietary fiber (g/day)	157	17.0 ± 5.4	133	17.9 ± 6.1
Saturated fatty acid (g/day)	157	21.4 ± 9.2	133	22.4 ± 8.4
Monounsaturated fatty acid (g/day)	157	23.1 ± 10.2	133	23.9 ± 9.3
Polyunsaturated fatty acid (g/day)	157	14.7 ± 5.4	133	15.3 ± 6.1
Cholesterol (mg/day)	157	336 ± 124	133	352 ± 129
Sodium (mg/day)	157	4826 ± 1767	133	4762 ± 1872
Potassium (mg/day)	157	2862 ± 946	133	3044 ± 1071
Calcium (mg/day)	157	767 ± 266	133	806 ± 282
Magnesium (mg/day)	157	298 ± 90	133	316 ± 106
Iron (mg/day)	157	9.5 ± 3.5	133	9.7 ± 3.5
Vitamin C (mg/day)	157	125 ± 58	133	130 ± 61
**Blood profiles**				
Triglyceride (mmol/l)	146	1.58 ± 1.14	117	1.48 ± 0.93
High-density lipoprotein cholesterol (mmol/l)	146	1.57 ± 0.45	117	1.61 ± 0.43
Low-density lipoprotein cholesterol (mmol/l)	146	3.15 ± 0.75	117	3.16 ± 0.71
Glycosylated hemoglobin A_1c_ (%)	146	5.67 ± 0.42	117	5.75 ± 0.63
Blood sugar (mmol/l)	146	6.08 ± 1.62	117	6.34 ± 1.51
Glutamic oxaloacetic transaminase (IU/l)	146	25.0 ± 6.4	117	24.9 ± 6.9
Glutamic pyruvic transaminase (IU/l)	146	19.8 ± 8.4	117	20.0 ± 7.4
γ-glutamyl transpeptidase (IU/l)	146	23.7 ± 15.1	117	27.7 ± 28.4
Albumin (g/l)	146	43.7 ± 2.7	117	43.8 ± 2.4
Creatinine (μmol/l)	146	67.5 ± 15.3	117	70.4 ± 16.0
Uric acid (μmol/l)	146	299 ± 77	117	298 ± 67
Estimated glomerular filtration rate (ml/min/1.73 m^2^)	146	65.9 ± 12.3	117	64.7 ± 12.7

LcS, *Lactobacillus casei* strain Shirota; SD, standard deviation. Intergroup differences in male/female ratio were assessed by chi-square tests. Independent differences in each of the other variables between groups were assessed by analyses of covariance, after adjusting data on age for sex and the others for age and sex. ^*^P < 0.05, ^∗∗^*P* < 0.01, and ^∗∗∗^*P* < 0.001, respectively, versus <15 min/day at >3 METs. Data on step count are not shown, mainly because of their similarities to those on the duration of exercise >3 METs.

**FIGURE 1:**
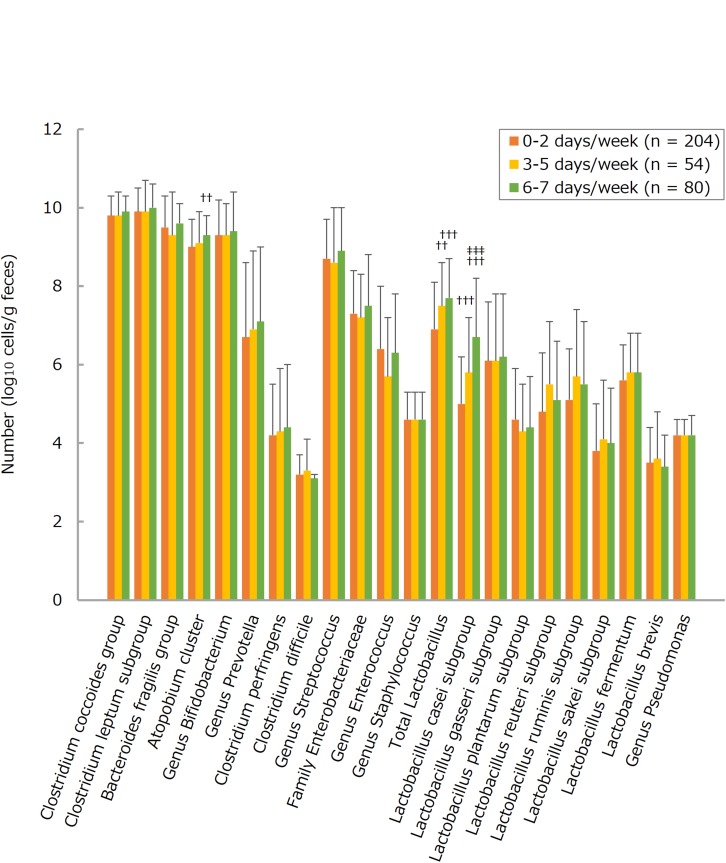
Numbers of fecal microbiota in subjects consuming fermented milk products containing *Lactobacillus casei* strain Shirota (LcS) 0–2, 3–5, or 6–7 days per week. Values are means ± standard deviations. Linear trends for three-group differences in each bacterial count were assessed by analyses of covariance, after adjusting data for age, sex, body mass index, smoking status, and alcohol intake; if the trends were statistically significant, *post hoc* Tukey’s tests assessed two-group differences in each bacterial count. ^†^^†^*P* < 0.01 and ^†^^†^^†^*P* < 0.001, respectively, versus 0–2 days/week; ^‡^^‡^^‡^*P* < 0.001 versus 3–5 days/week. Data on overall fermented milk products are not shown, mainly because of their similarities to those on LcS-containing products.

**FIGURE 2:**
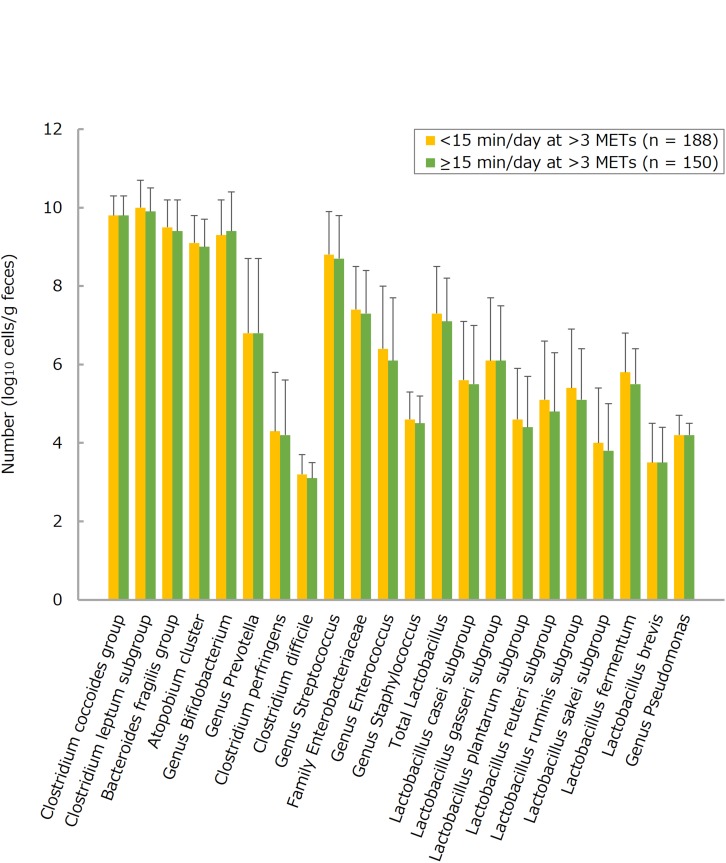
Numbers of fecal microbiota in subjects taking exercise demanding an energy expenditure >3 metabolic equivalents (METs) for <15 or ≥15 min per day. Values are means ± standard deviations. Independent differences in each bacterial count between groups were assessed by analyses of covariance, after adjusting data for age, sex, body mass index, smoking status, and alcohol intake. Data on step count are not shown, mainly because of their similarities to those on the duration of exercise >3 METs.

**FIGURE 3:**
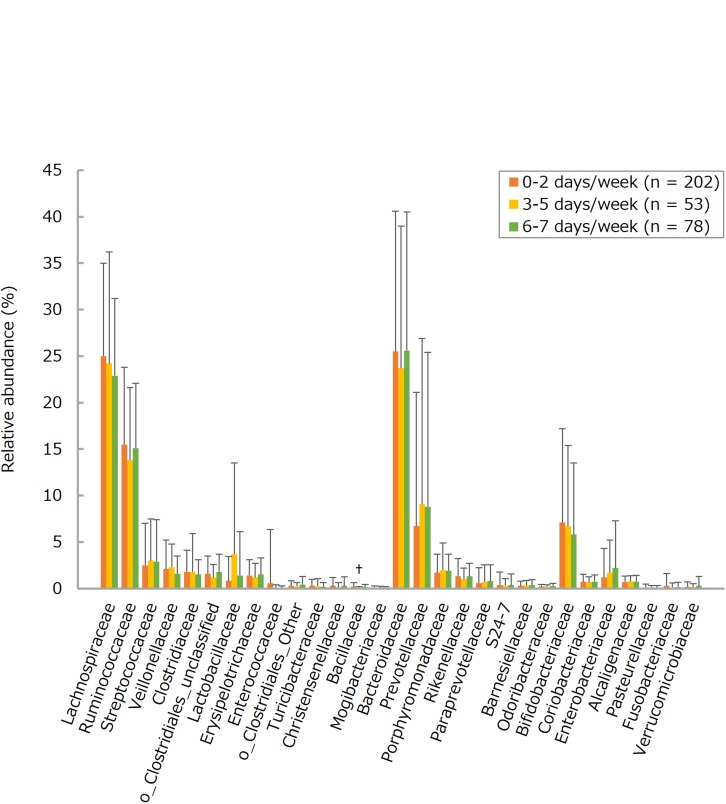
Relative abundances of the fecal bacteria families (limited to a share ≥0.1%) in subjects consuming fermented milk products containing *Lactobacillus casei* strain Shirota (LcS) 0–2, 3–5, or 6–7 days per week. Values are means ± standard deviations. Linear trends for three-group differences in each bacterial abundance were assessed by analyses of covariance, after adjusting data for age, sex, body mass index, smoking status, and alcohol intake; if the trends were statistically significant, *post hoc* Tukey’s tests assessed two-group differences in each bacterial abundance. ^†^*P* < 0.05 versus 0–2 days/week. Data on overall fermented milk products are not shown, mainly because of their similarities to those on LcS-containing products.

**FIGURE 4:**
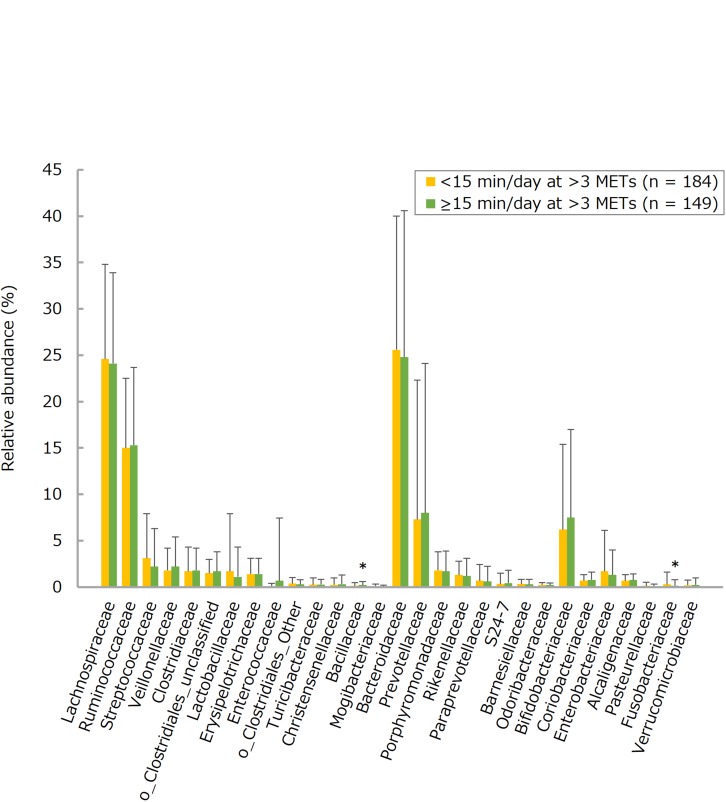
Relative abundances of the fecal bacteria families (limited to a share ≥0.1%) in subjects taking exercise demanding an energy expenditure >3 metabolic equivalents (METs) for <15 or ≥15 min per day. Values are means ± standard deviations. Independent differences in each bacterial abundance between groups were assessed by analyses of covariance, after adjusting data for age, sex, body mass index, smoking status, and alcohol intake. ^*^P < 0.05 versus <15 min/day at >3 METs. Data on step count are not shown, mainly because of their similarities to those on the duration of exercise >3 METs.

The number of each of many bacteria detected in the intestines tended to be larger in the more frequent consumers of LcS-containing products, this trend being statistically significant for total *Lactobacillus* (groups 0–2 versus 3–5 and 6–7), the *Lactobacillus casei* subgroup (groups 0–2 versus 3–5 and 6–7; groups 3–5 versus 6–7), and the *Atopobium* cluster (groups 0–2 versus 6–7) (Figure 1 and Supplementary Table 5). On the other hand, there were no statistically significant differences between <15 and ≥15 physical activity groups with respect to counts for any of the selected fecal bacteria (Figure 2 and Supplementary Table 6). Furthermore, there were no significant intergroup differences in the relative abundance of any of the fecal bacteria families (limited to a share ≥0.1%) except Bacillaceae (LcS groups 0–2 versus 3-5; activity groups <15 versus ≥15) and Fusobacteriaceae (activity groups <15 versus ≥15) (Figures 3, 4 and Supplementary Tables 7, 8).

Our population in general had regular and normal bowel movements, and in terms of the intake of both LcS and overall fermented milk products, no significant intergroup differences were observed in either the frequency of defecation or the consistency of the stools (Table 3). On the other hand, in terms of habitual physical activity, the frequency of defecation differed significantly both between <7000 and ≥7000 groups and between <15 and ≥15 groups, although there were no significant intergroup differences in BSFS rating (Table 4).

**TABLE 3:** Defecation frequency and scores on the Bristol Stool Form Scale (BSFS) in subjects consuming fermented milk products containing *Lactobacillus casei* strain Shirota (LcS) and overall fermented milk products 0–2, 3–5, or 6–7 days per week.

	**Defecation frequency**		**BSFS score**	
		
	***n***	**Mean ± SD**	***n***	**Mean ± SD**
		**(days/week)**		
**LcS fermented milk products**				
0–2 days/week	204	5.9 ± 1.7	201	4.0 ± 0.9
3–5 days/week	54	6.0 ± 1.7	54	4.0 ± 1.0
6–7 days/week	80	5.9 ± 1.6	80	4.2 ± 0.9
**Overall fermented milk products**				
0–2 days/week	78	5.8 ± 1.8	76	4.1 ± 0.9
3–5 days/week	70	6.0 ± 1.6	70	3.9 ± 0.8
6–7 days/week	190	6.0 ± 1.7	189	4.0 ± 1.0

*SD, standard deviation. Independent differences in each variable among groups were assessed by analyses of covariance, after adjusting data for age, sex, body mass index, smoking status, and alcohol intake.*

**TABLE 4:** Defecation frequency and scores on the Bristol Stool Form Scale (BSFS) in subjects taking <7000 or ≥7000 steps per day and spending <15 or ≥15 min per day of exercise at an intensity >3 metabolic equivalents (METs).

	**Defecation frequency**		**BSFS score**	
		
	***n***	**Mean ± SD**	***n***	**Mean ± SD**
		**(days/week)**		
**Step count**				
<7000 steps/day	187	5.6 ± 1.9	185	4.0 ± 1.0
≥7000 steps/day	151	6.3 ± 1.3^∗∗^	150	4.0 ± 0.8
**Duration of exercise >3 METs**				
<15 min/day at >3 METs	188	5.6 ± 1.9	187	4.0 ± 1.0
≥15 min/day at >3 METs	150	6.3 ± 1.3^∗∗^	148	4.0 ± 0.8

*SD, standard deviation. Independent differences in each variable between groups were assessed by analyses of covariance, after adjusting data for age, sex, body mass index, smoking status, and alcohol intake. ^∗∗^*P* < 0.01 versus <7000 steps/day or <15 min/day at >3 METs.*

Relating defecation to LcS and overall supplement consumption, respectively, 29/204 (14.2%) and 14/78 (17.9%) of group 0–2, 5/54 (9.3%) and 8/70 (11.4%) of group 3–5, and 7/80 (8.8%) and 19/190 (10.0%) of group 6–7 had bowel movements ≤3 days/week (Table 5); the 5% and 8% differences in the prevalence of infrequent bowel movements between 0–2 and 6–7 groups became statistically significant after multivariate adjustment, with a substantial advantage to group 6–7 [respective odds ratios (95% confidence intervals) of 0.382 (0.149–0.974) and 0.314 (0.134–0.732)] relative to group 0–2 (Table 5). On the other hand, in terms of habitual physical activity, the analogous statistically significant figures were 10% and 11% [31/187 (16.6%) of group <7000 versus 10/151 (6.6%) of group ≥7000 and 32/188 (17.0%) of group <15 versus 9/150 (6.0%) of group ≥15, respectively], and 0.441 (0.201–0.971) in group ≥7000 relative to group <7000 and 0.412 (0.183–0.929) in group ≥15 relative to group <15 (Table 6).

**TABLE 5:** Estimated risks of developing infrequent bowel movements in subjects consuming fermented milk products containing *Lactobacillus casei* strain Shirota (LcS) and overall fermented milk products 0–2, 3–5, or 6–7 days per week.

	***n***	**Infrequent bowel movements rate (%)**	**Odds ratio**	**95% confidence interval**
**LcS fermented milk products**				
0–2 days/week	204	14.2	1	–
3–5 days/week	54	9.3	0.493	0.174–1.397
6–7 days/week	80	8.8	0.382^†^	0.149–0.974
**Overall fermented milk products**				
0–2 days/week	78	17.9	1	–
3–5 days/week	70	11.4	0.514	0.192–1.380
6–7 days/week	190	10.0	0.314^††^	0.134–0.732

*Intergroup differences in the rate of infrequent bowel movements were assessed by chi-square tests. Odds ratios and 95% confidence intervals were determined by logistic regression analyses, after adjusting data for age, sex, body mass index, smoking status, and alcohol intake. ^†^*P* < 0.05 and ^†^^†^*P* < 0.01, respectively, versus 0–2 days/week.*

**TABLE 6:** Estimated risks of developing infrequent bowel movements in subjects taking <7000 or ≥7000 steps per day and spending <15 or ≥15 min per day of exercise at an intensity >3 metabolic equivalents (METs).

	***n***	**Infrequent bowel movements rate (%)**	**Odds ratio**	**95% confidence interval**
**Step count**				
<7000 steps/day	187	16.6	1	–
≥7000 steps/day	151	6.6^∗∗^	0.441^*^	0.201–0.971
**Duration of exercise >3 METs**				
<15 min/day at >3 METs	188	17.0	1	–
≥15 min/day at >3 METs	150	6.0^∗∗^	0.412^*^	0.183–0.929

Intergroup differences in the rate of infrequent bowel movements were assessed by chi-square tests. Odds ratios and 95% confidence intervals were determined by logistic regression analyses, after adjusting data for age, sex, body mass index, smoking status, and alcohol intake. ^*^P < 0.05 and ^∗∗^*P* < 0.01, respectively, versus <7000 steps/day or <15 min/day at >3 METs.

In terms of interactions between fermented milk product intakes and habitual physical activity patterns, there were tendencies to differences in the rate of infrequent bowel movements between combinations of variables (significantly so for 0–2 days/week × <7000 steps/day or <15 min/day at >3 METs versus some other combinations), although a small proportion of individuals taking LcS or overall fermented milk products 6–7 days/week and engaging in ≥7000 steps/day or exercise >3 METs for ≥15 min/day also had such bowel problems (Table 7). After multivariate adjustment, the estimated risks of demonstrating infrequent bowel movements tended to be lower if the frequency of supplement ingestion and/or the level of physical activity were/was greater. Using the least frequent and physically less active users of the products as the comparison group, the risk of such bowel problems was no more than one-fifth as large for subjects who consumed LcS-containing fermented milk products 6–7 days/week and took ≥7000 steps/day or spent <15 min/day of physical activity >3 METs, and it dropped to about one-tenth for those consuming overall fermented milk products 6–7 days/week and taking ≥7000 steps/day or spending both <15 and ≥15 min/day at >3 METs (Table 7).

**TABLE 7:** Interactions between fermented milk product intakes and habitual physical activity patterns in terms of estimated risks of developing infrequent bowel movements in sample of 338 elderly Japanese.

		***n***	**Infrequent bowel movements rate (%)**	**Odds ratio**	**95% confidence interval**
**LcS fermented milk products × step count**					
0–2 days/week	<7000 steps/day	112	18.8	1	–
	≥7000 steps/day	92	8.7^§^	0.432	0.173–1.080
3–5 days/week	<7000 steps/day	34	11.8	0.410	0.119–1.419
	≥7000 steps/day	20	5.0	0.181	0.021–1.536
6-7 days/week	<7000 steps/day	41	14.6	0.327	0.104–1.028
	≥7000 steps/day	39	2.6^§^	0.121^§^	0.015–0.964
**LcS fermented milk products × duration of exercise >3 METs**					
0–2 days/week	<15 min/day at >3 METs	108	19.4	1	–
	≥15 min/day at >3 METs	96	8.3^§^	0.428	0.173–1.060
3–5 days/week	<15 min/day at >3 METs	37	13.5	0.477	0.154–1.477
	≥15 min/day at >3 METs	17	0.0^§^	0.000	0.000–infinity
6–7 days/week	<15 min/day at >3 METs	43	14.0	0.306^§^	0.098–0.953
	≥15 min/day at >3 METs	37	2.7^§^	0.126	0.016–1.012
**Overall fermented milk products × step count**					
0–2 days/week	<7000 steps/day	46	21.7	1	–
	≥7000 steps/day	32	12.5	0.438	0.105–1.836
3–5 days/week	<7000 steps/day	36	11.1	0.351	0.086–1.431
	≥7000 steps/day	34	11.8	0.431	0.110–1.688
6–7 days/week	<7000 steps/day	105	16.2	0.371	0.132–1.044
	≥7000 steps/day	85	2.4^§§§^	0.078^§§^	0.015–0.417
**Overall fermented milk products × duration of exercise >3 METs**					
0–2 days/week	<15 min/day at >3 METs	43	23.3	1	–
	≥15 min/day at >3 METs	35	11.4	0.437	0.111–1.727
3–5 days/week	<15 min/day at >3 METs	40	12.5	0.282	0.074–1.068
	≥15 min/day at >3 METs	30	10.0	0.315	0.071–1.394
6–7 days/week	<15 min/day at >3 METs	105	16.2	0.310^§^	0.109–0.878
	≥15 min/day at >3 METs	85	2.4^§§§^	0.065^§§^	0.012–0.344

*Intercombination differences in the rate of infrequent bowel movements were assessed by chi-square tests. Odds ratios and 95% confidence intervals were determined by logistic regression analyses, after adjusting data for age, sex, body mass index, smoking status, and alcohol intake. ^§^*P* < 0.05, ^§§^*P* < 0.01, and ^§§§^*P* < 0.001, respectively, versus 0–2 days/week × <7000 steps/day or <15 min/day at >3 METs.*

## Discussion

Clinical investigations to date have generally shown that either the daily intake of fermented milk products containing LcS (Matsumoto et al., 2006, 2010; Shioiri et al., 2006; Sakai et al., 2011) or regular engagement in physical activity or exercise (Sandler et al., 1990; Dukas et al., 2003; De Schryver et al., 2005) augments intestinal motility and/or induces favorable changes in the local microbiotal environment, and that this is associated with a reduced risk of infrequent bowel movements in young and middle-aged people. However, since the extent of the individual’s habitual physical activity has been estimated by relatively inaccurate questionnaires, the impact of LcS and objectively measured physical activity on such parameters remains to be clearly defined in healthy community-dwelling individuals. The present cross-sectional epidemiological study supports other clinical observations (Sandler et al., 1990; Dukas et al., 2003; De Schryver et al., 2005; Matsumoto et al., 2006, 2010; Shioiri et al., 2006; Sakai et al., 2011) in showing an association between the frequent ingestion of LcS or the regular performance of physical activity and a reduced risk of infrequent bowel movements. The new data confirm that in elderly people with no serious health problems, the likelihood of reporting infrequent bowel movements is lower in those with a higher habitual intake of LcS-containing fermented milk products and/or a greater quantity/quality of habitual physical activity.

There seems a tendency toward finding greater respective numbers of gut microbiota, especially total *Lactobacillus* and the *Lactobacillus casei* subgroup, as the frequency of ingesting LcS-containing fermented milk products is increased. Many clinical trials (Yuki et al., 1999; Matsumoto et al., 2006; Sakai et al., 2011; Suzuki et al., 2017) have also shown that a large fraction of the ingested population of LcS survives in the human gastrointestinal tract; this may be attributed to a high tolerance of LcS to gastric and bile acids (Kobayashi et al., 1974). In our subjects, most of whom had regular and normal bowel movements (on average about 6 days/week; an average BSFS score of about 4), no increases in the frequency of defecation or no improvements in the consistency of stool were seen in those ingesting LcS-containing supplements more frequently. Nevertheless, the likelihood of infrequent bowel movements (defined as defecating ≤3 days/week) was lowest in elderly individuals who consumed LcS-containing fermented milk products 6–7 days/week. In consequence, the estimated risk of such bowel problems in these individuals was about one-third [a significant odds ratio (95% confidence interval) of 0.382 (0.149–0.974)] after controlling data for potential confounders. These findings support the suggestion (as evaluated by a randomized, double-blind, placebo-controlled, crossover scheme; Matsumoto et al., 2006) that almost daily consumption of LcS-containing fermented milk products could benefit older people who tend to be constipated by increasing the intestinal population of beneficial microbes (*Lactobacillus* species) and raising the intestinal level of metabolites of microbial fermentation (lactic acids), thus stimulating intestinal peristalsis/motility (Matsumoto, 2013; Mai et al., 2017).

On the other hand, habitual physical activity (whether expressed as daily step count or daily duration at an intensity >3 METs) was not associated with the number of any of the fecal bacteria analyzed in the present investigation. However, there was a small but statistically significant difference of mean defecation frequency (<1 day/week) between physically more and less active individuals, irrespective of whether physical activity was expressed as daily step count or the duration of activity >3 METs. The prevalence of infrequent bowel movements was some ≥10% lower in elderly people who took ≥7000 steps/day or spent ≥15 min/day of physical activity >3 METs than in those with <7000 steps/day or <15 min/day of activity >3 METs. Further, the multivariate-adjusted estimate of the risk of such bowel problems was >50% smaller for study participants with a physically more active lifestyle as compared to those who were less active. Therefore, it appears that in contrast to LcS, moderate daily physical activity reduces the risk of infrequent bowel movements mainly by a mechanical stimulation of intestinal motions, without changing gut bacterial counts. Nevertheless, other factors could also be involved, including transient changes in blood flow to the gastrointestinal tract, altered levels of circulating hormones, and changes in the balance between sympathetic and parasympathetic innervation (Oettlé, 1991; Rao et al., 1999; Peters et al., 2001; De Schryver et al., 2005).

The present investigation confirmed statistically significant interactions between infrequent bowel movements, the ingestion of fermented milk products (LcS or overall supplements) and patterns of habitual physical activity (step count or duration at >3 METs). Using the least frequent and physically less active consumers of these products (0–2 days/week × <7000 steps/day or <15 min/day at >3 METs) as the reference group, the likelihood of such bowel problems was only a tenth as great in individuals who ingested LcS or overall fermented milk products 6–7 days/week and took ≥7000 steps/day or spent ≥15 min/day of activity >3 METs. These results suggest that to optimize intestinal health and to minimize the risk of infrequent bowel movements, elderly individuals could be encouraged to ingest a fermented milk supplement such as LcS as regularly as possible (at least 6 days/week) and to engage in moderate habitual physical activity (at least 7000 steps/day and/or at least 15 min/day at >3 METs).

There are some limitations to the current investigation. The design was cross-sectional and observational rather than longitudinal and experimental, so that causation cannot be inferred. The estimated frequencies of LcS intake and bowel movement were based on questionnaires administered regularly and repeatedly by the subjects, using a standardized methodology. Furthermore, physical activity patterns were automatically recorded for 24 h/day, using the pedometer/accelerometer, as part of the ongoing Nakanojo Study (Aoyagi and Shephard, 2009, 2010, 2011, 2013), and since the main activity of this age group was walking, energy expenditures were estimated fairly accurately, the main exception being the effort expended in walking uphill. These facts strengthen the practical significance of observed relationships between the frequency of LcS-containing product consumption, the pattern of habitual physical activity and the risk of developing infrequent bowel movements. On the other hand, individuals ingesting fermented milk supplements almost every day and/or taking ≥7000 steps/day and/or spending ≥15 min/day of physical activity >3 METs might differ from their peers in terms of a greater overall interest in personal lifestyle, embracing other facets of healthy living that could reduce their risk of infrequent bowel movements. Many lifestyle covariates were examined; those who ingested fermented milk products almost every day were older and somewhat better nourished, and those who engaged in moderate habitual daily physical activity were younger, less fat, and faster walkers than their peers, but in most respects, the various groups of study participants appeared to be well-matched. Further, we co-varied for the most important lifestyle determinants of infrequent bowel movements (age, sex, body mass index, cigarette smoking, and alcohol consumption), although the statistical adjustment for these factors may have been less than complete. People typically drink the entire bottle, but the amount of LcS-containing fermented milk ingested is not necessarily related to the frequency of its ingestion. Moreover, the risk of becoming constipated is linked to many other aspects of food intake, in particular dietary fiber content, and the response to a frequent intake of LcS-containing fermented milk might diverge in a population whose diet differed substantially from the rice-based nutrition of the elderly in rural Japan. The etiology of constipation also differs between young and old people, so there is a need for this study to be replicated in populations of various ages and consuming various diets.

## Conclusion

After adjustment for potential confounders, the risk of developing infrequent bowel movements in older people is inversely associated with the frequency of ingestion of fermented milk products containing LcS and with the quantity and quality of habitual physical activity. The risk of such bowel problems is substantially lower for seniors who consume such products 6–7 days/week and/or take ≥7000 steps/day and/or spend ≥15 min/day of physical activity at an intensity demanding an energy expenditure >3 METs. The main mechanism for enhancing bowel function appears to differ between LcS supplementation (an increase of intestinal lactobacilli) and physical activity (a mechanical stimulation of intestinal movements). Nevertheless, interventional studies are needed to determine whether there is a cause-and-effect relationship between these three variables.

## Ethics Statement

This study was carried out in accordance with the recommendations of the ethics review committee of the Tokyo Metropolitan Institute of Gerontology with written informed consent from all subjects. All subjects gave written informed consent in accordance with the Declaration of Helsinki. The protocol was approved by the ethics review committee of the Tokyo Metropolitan Institute of Gerontology.

## Author Contributions

YA contributed to the study concept and design, acquisition of subjects and data, analysis and interpretation of data, and preparation of manuscript. RA, YH, KaS, AK, HT, HM, KeS, KM, and SM contributed to assay of gut microbiota. SP acquired the subjects and data and analyzed the data. RS interpreted the data and prepared the manuscript.

## Conflict of Interest Statement

RA, YH, KaS, AK, HT, HM, KeS, KM, and SM are affiliated with the Yakult Honsha Co., Ltd. The funders had no role in study design, data collection and analysis, decision to publish, or preparation of the manuscript. The remaining authors declare that the research was conducted in the absence of any commercial or financial relationships that could be construed as a potential conflict of interest.
