# Supplementary material for: Independent and Interactive Effects of Habitually Ingesting Fermented Milk Products Containing Lactobacillus casei Strain Shirota and of Engaging in Moderate Habitual Daily Physical Activity on the Intestinal Health of Older People
Source: Front Microbiol. 2019 Jul 31;10:1477. doi: 10.3389/fmicb.2019.01477 (PMC6684969; doi:10.3389/fmicb.2019.01477)
Supplement: Supplementary file 1 [file Table_1.docx]

***Supplementary Material***

**Independent and interactive effects of habitually ingesting fermented milk products containing *Lactobacillus casei* strain Shirota and of engaging in moderate habitual daily physical activity on the intestinal health of older people**

**Yukitoshi Aoyagi**^1*^**, Ryuta Amamoto**^2^**, Sungjin Park**^1^**, Yusuke Honda**^2^**, Kazuhito Shimamoto**^2^**, Akira Kushiro**^3^**, Hirokazu Tsuji**^4^**, Hoshitaka Matsumoto**^3^**, Kensuke Shimizu**^3^**, Kouji Miyazaki**^2^**, Satoshi Matsubara**^2^ **and Roy J. Shephard**^5^

^1^Exercise Sciences Research Group, Tokyo Metropolitan Institute of Gerontology, Itabashi, Tokyo, Japan

^2^Food Research Department, Yakult Central Institute, Kunitachi, Tokyo, Japan

^3^Microbiological Research Department, Yakult Central Institute, Kunitachi, Tokyo, Japan

^4^Basic Research Department, Yakult Central Institute, Kunitachi, Tokyo, Japan

^5^Faculty of Kinesiology and Physical Education, University of Toronto, Toronto, ON, Canada

**^*^Correspondence:** Yukitoshi Aoyagi, [aoyagi@tmig.or.jp](mailto:aoyagi@tmig.or.jp)

**Supplementary Table 1.** Frequency distribution of consumption of fermented milk products containing *Lactobacillus casei* strain Shirota (LcS) and overall fermented milk products in sample of 338 elderly Japanese

|  | Intake frequency (days/week) | | | | | | | |
| --- | --- | --- | --- | --- | --- | --- | --- | --- |
|  | 0 | 1 | 2 | 3 | 4 | 5 | 6 | 7 |
| LcS fermented milk products | 143 | 33 | 28 | 19 | 13 | 22 | 13 | 67 |
| Overall fermented milk products | 40 | 23 | 15 | 30 | 13 | 27 | 26 | 164 |

**Supplementary Table 2.** Frequency distribution of daily step count and daily duration of exercise at an intensity >3 metabolic equivalents (METs) in sample of 338 elderly Japanese

|  | Physical activity category | | | | |
| --- | --- | --- | --- | --- | --- |
|  | C1 | C2 | C3 | C4 | C5 |
| Step count | 11 | 78 | 98 | 75 | 76 |
| Duration of exercise >3 METs | 28 | 65 | 95 | 68 | 82 |

C1-C5, first through fifth categories of habitual daily physical activity in elderly people, based on data from the Nakanojo Study (Aoyagi and Shephard, 2009, 2010, 2011, 2013). Older adults are classified on the bases of step count and/or the duration of exercise >3 METs:

C1 = <2000 steps/day and/or <2.5 min/day at >3 METs;

C2 = 2000-<5000 (mean 4000) steps/day and/or 2.5-<7.5 (mean 5) min/day at >3 METs;

C3 = 5000-<7000 (mean 6000) steps/day and/or 7.5-<15 (mean 10) min/day at >3 METs;

C4 = 7000-<9000 (mean 8000) steps/day and/or 15-<25 (mean 20) min/day at >3 METs;

C5 = ≥9000 (mean 10000) steps/day and/or ≥25 (mean 30) min/day at >3 METs.

**Supplementary Table 3.** Primer sequences (5’-3’), annealing temperatures and minimum detection limits for quantification of selected gut bacteria

| Primer sequence | Annealing temperature (°C) | Detection limit  (log_10_ cells/g feces) | Reference |
| --- | --- | --- | --- |
| *Clostridium coccoides* group |  | 5.0 |  |
| Forward: AAATGACGGTACCTGACTAA | 55 |  | Matsuki et al., 2004 |
| Reverse: CTTTGAGTTTCATTCTTGCGAA |  |  |  |
| *Clostridium leptum* subgroup |  | 4.1 |  |
| Forward: GCACAAGCAGTGGAGT | 55 |  | Matsuki et al., 2004 |
| Reverse: CTTCCTCCGTTTTGTCAA |  |  |  |
| *Bacteroides fragilis* group |  | 4.0 |  |
| Forward: AYAGCCTTTCGAAAGRAAGAT | 50 |  | Matsuki, 2007 |
| Reverse: CCAGTATCAACTGCAATTTTA |  |  |  |
| *Atopobium* cluster |  | 5.1 |  |
| Forward: GGGTTGAGAGACCGACC | 55 |  | Matsuki et al., 2004 |
| Reverse: CGGRGCTTCTTCTGCAGG |  |  |  |
| Genus *Bifidobacterium* |  | 5.0 |  |
| Forward: CTCCTGGAAACGGGTGG | 55 |  | Matsuki et al., 2004 |
| Reverse: GGTGTTCTTCCCGATATCTACA |  |  |  |
| Genus *Prevotella* |  | 5.2 |  |
| Forward: CACRGTAAACGATGGATGCC | 55 |  | Matsuki et al., 2004 |
| Reverse: GGTCGGGTTGCAGACC |  |  |  |
| *Clostridium perfringens* |  | 3.2 |  |
| Forward: GGGGGTTTCAACACCTCC | 60 |  | Matsuda et al., 2009 |
| Reverse: GCAAGGGATGTCAAGTGT |  |  | Kikuchi et al., 2002 |
| *Clostridium difficile* |  | 3.1 |  |
| Forward: GGGAGCTTCCCATACGGGTTG | 60 |  | Matsuda et al., 2012 |
| Reverse: TTGACTGCCTCAATGCTTGGGC |  |  |  |
| Genus *Streptococcus* |  | 4.1 |  |
| Forward: AGCTTAGAAGCAGCTATTCATTC | 60 |  | Sakaguchi et al., 2010 |
| Reverse: GGATACACCTTTCGGTCTCTC |  |  |  |
| Family Enterobacteriaceae |  | 5.2 |  |
| Forward: TGCCGTAACTTCGGGAGAAGGCA | 60 |  | Matsuda et al., 2007 |
| Reverse: TCAAGGACCAGTGTTCAGTGTC |  |  |  |
| Genus *Enterococcus* |  | 4.1 |  |
| Forward: ATCAGAGGGGGATAACACTT | 55 |  | Matsuda et al., 2009 |
| Reverse: ACTCTCATCCTTGTTCTTCTC |  |  |  |
| Genus *Staphylococcus* |  | 4.1 |  |
| Forward: TTTGGGCTACACACGTGCTACAATGGACAA | 60 |  | Matsuda et al., 2009 |
| Reverse: AACAACTTTATGGGATTTGCWTGA |  |  |  |
| *Lactobacillus casei* subgroup |  | 4.0 |  |
| Forward: ACCGCATGGTTCTTGGC | 60 |  | Matsuda et al., 2009 |
| Reverse: CCGACAACAGTTACTCTGCC |  |  |  |
| *Lactobacillus gasseri* subgroup |  | 3.1 |  |
| Forward: GATGCATAGCCGAGTTGAGAGACTGAT | 60 |  | Matsuda et al., 2009 |
| Reverse: TAAAGGCCAGTTACTACCTCTATCC |  |  |  |
| *Lactobacillus plantarum* subgroup |  | 3.2 |  |
| Forward: CTCTGGTATTGATTGGTGCTTGCAT | 60 |  | Matsuda et al., 2009 |
| Reverse: GTTCGCCACTCACTCAAATGTAAA |  |  |  |
| *Lactobacillus reuteri* subgroup |  | 3.2 |  |
| Forward: GAACGCAYTGGCCCAA | 60 |  | Matsuda et al., 2009 |
| Reverse: TCCATTGTGGCCGATCAGT |  |  |  |
| *Lactobacillus ruminis* subgroup |  | 4.4 |  |
| Forward: CACCGAATGCTTGCAYTCACC | 60 |  | Matsuda et al., 2009 |
| Reverse: GCCGCGGGTCCATCCAAAA |  |  |  |
| *Lactobacillus sakei* subgroup |  | 3.1 |  |
| Forward: CATAAAACCTAMCACCGCATGG | 60 |  | Matsuda et al., 2009 |
| Reverse: TCAGTTACTATCAGATACRTTCTTCTC |  |  |  |
| *Lactobacillus fermentum* |  | 5.1 |  |
| Forward: CCTGATTGATTTTGGTCGCCAAC | 55 |  | Watanabe, 1998 |
| Reverse: ACGTATGAACAGTTACTCTCATACGT |  |  |  |
| *Lactobacillus brevis* |  | 3.1 |  |
| Forward: ATTTTGTTTGAAAGGTGGCTTCGG | 55 |  | Matsuda et al., 2009 |
| Reverse: ACCCTTGAACAGTTACTCTCAAAGG |  |  |  |
| Genus *Pseudomonas* |  | 4.1 |  |
| Forward: CAAAACTACTGAGCTAGAGTACG | 60 |  | Matsuda et al., 2007 |
| Reverse: TAAGATCTCAAGGATCCCAACGGCT |  |  |  |

Y, T or C; R, A or G; W, A or T; M, A or C.

**Supplementary Table 4.** Primer sequences (5’-3’) and annealing temperatures for amplification of the 16S rRNA gene of gut bacteria

| Primer sequence | Annealing  temperature (°C) | Reference |
| --- | --- | --- |
| Forward (27Fmod2-MiSeqV2):  AATGATACGGCGACCACCGAGATCTACACTCTTTCCCTA-  CACGACGCTCTTCCGATCTAGRGTTYGATYMTGGCTCAG | 55 | Kato-Kataoka et al., 2016 |
| Reverse (338RMiSeqV2-001):  CAAGCAGAAGACGGCATACGAGATNNNNNNNNNNGTGACTGG-  AGTTCAGACGTGTGCTCTTCCGATCTGCTGCCWCCCGTAGGWGT |  |  |

R, A or G; Y, T or C; M, A or C; W, A or T.

NNNNNNNNNN represents the unique bar-code sequence for tagging each PCR product.

**Supplementary Table 5.** Numbers of fecal microbiota in subjects consuming fermented milk products containing *Lactobacillus casei* strain Shirota (LcS) 0-2, 3-5 or 6-7 days per week

|  | 0-2 days/week (n = 204) | | | |  | 3-5 days/week (n = 54) | | | |  | 6-7 days/week (n = 80) | | | |
| --- | --- | --- | --- | --- | --- | --- | --- | --- | --- | --- | --- | --- | --- | --- |
|  | Detection rate (%) | Mean ± SD  (log_10_ cells/  g feces) | | |  | Detection rate (%) | Mean ± SD  (log_10_ cells/  g feces) | | |  | Detection rate (%) | Mean ± SD  (log_10_ cells/  g feces) | | |
| Obligate anaerobes |  |  |  |  |  |  |  |  |  |  |  |  |  |  |
| *Clostridium coccoides* group | 100 | 9.8 | ± | 0.5 |  | 100 | 9.8 | ± | 0.6 |  | 100 | 9.9 | ± | 0.4 |
| *Clostridium leptum* subgroup | 100 | 9.9 | ± | 0.6 |  | 100 | 9.9 | ± | 0.8 |  | 100 | 10.0 | ± | 0.6 |
| *Bacteroides fragilis* group | 100 | 9.5 | ± | 0.8 |  | 98 | 9.3 | ± | 1.1 |  | 100 | 9.6 | ± | 0.5 |
| *Atopobium* cluster | 100 | 9.0 | ± | 0.7 |  | 100 | 9.1 | ± | 0.8 |  | 100 | 9.3 | ± | 0.5 †† |
| Genus *Bifidobacterium* | 99 | 9.3 | ± | 0.9 |  | 100 | 9.3 | ± | 0.8 |  | 100 | 9.4 | ± | 1.0 |
| Genus *Prevotella* | 65 | 6.7 | ± | 1.9 |  | 69 | 6.9 | ± | 2.0 |  | 71 | 7.1 | ± | 1.9 |
| *Clostridium perfringens* | 50 | 4.2 | ± | 1.3 |  | 50 | 4.3 | ± | 1.6 |  | 53 | 4.4 | ± | 1.6 |
| *Clostridium difficile* | 3 | 3.2 | ± | 0.5 |  | 7 | 3.3 | ± | 0.8 |  | 3 | 3.1 | ± | 0.1 |
| Facultative anaerobes |  |  |  |  |  |  |  |  |  |  |  |  |  |  |
| Genus *Streptococcus* | 99 | 8.7 | ± | 1.0 |  | 94 | 8.6 | ± | 1.4 |  | 98 | 8.9 | ± | 1.1 |
| Family Enterobacteriaceae | 97 | 7.3 | ± | 1.1 |  | 94 | 7.2 | ± | 1.1 |  | 93 | 7.5 | ± | 1.3 |
| Genus *Enterococcus* | 85 | 6.4 | ± | 1.6 |  | 69 | 5.7 | ± | 1.5 |  | 83 | 6.3 | ± | 1.5 |
| Genus *Staphylococcus* | 53 | 4.6 | ± | 0.7 |  | 52 | 4.6 | ± | 0.7 |  | 61 | 4.6 | ± | 0.7 |
| Total *Lactobacillus* | 99 | 6.9 | ± | 1.2 |  | 100 | 7.5 | ± | 1.1 †† |  | 100 | 7.7 | ± | 1.0 ††† |
| *Lactobacillus casei* subgroup | 52 | 5.0 | ± | 1.2 |  | 81 | 5.8 | ± | 1.4 ††† |  | 86 | 6.7 | ± | 1.5 †††, ‡‡‡ |
| *Lactobacillus gasseri* subgroup | 93 | 6.1 | ± | 1.5 |  | 87 | 6.1 | ± | 1.7 |  | 95 | 6.2 | ± | 1.6 |
| *Lactobacillus plantarum* subgroup | 75 | 4.6 | ± | 1.3 |  | 67 | 4.3 | ± | 1.2 |  | 74 | 4.4 | ± | 1.3 |
| *Lactobacillus reuteri* subgroup | 75 | 4.8 | ± | 1.5 |  | 85 | 5.5 | ± | 1.6 |  | 80 | 5.1 | ± | 1.5 |
| *Lactobacillus ruminis* subgroup | 34 | 5.1 | ± | 1.3 |  | 56 | 5.7 | ± | 1.7 |  | 41 | 5.5 | ± | 1.6 |
| *Lactobacillus sakei* subgroup | 34 | 3.8 | ± | 1.2 |  | 46 | 4.1 | ± | 1.5 |  | 38 | 4.0 | ± | 1.4 |
| *Lactobacillus fermentum* | 39 | 5.6 | ± | 0.9 |  | 48 | 5.8 | ± | 1.0 |  | 49 | 5.8 | ± | 1.0 |
| *Lactobacillus brevis* | 33 | 3.5 | ± | 0.9 |  | 30 | 3.6 | ± | 1.2 |  | 29 | 3.4 | ± | 0.8 |
| Aerobes |  |  |  |  |  |  |  |  |  |  |  |  |  |  |
| Genus *Pseudomonas* | 11 | 4.2 | ± | 0.4 |  | 11 | 4.2 | ± | 0.4 |  | 15 | 4.2 | ± | 0.5 |

SD, standard deviation.

Linear trends for three-group differences in each bacterial count were assessed by analyses of covariance, after adjusting data for age, sex, body mass index, smoking status and alcohol intake; if the trends were statistically significant, *post hoc* Tukey’s tests assessed two-group differences in each bacterial count.

†† and ††† *P* < 0.01 and *P* < 0.001, respectively, versus 0-2 days/week; ‡‡‡ *P* < 0.001 versus 3-5 days/week.

Data on overall fermented milk products are not shown, mainly because of their similarities to those on LcS-containing products.

**Supplementary Table 6.** Numbers of fecal microbiota in subjects taking exercise demanding an energy expenditure >3 metabolic equivalents (METs) for <15 or ≥15 min per day

|  | <15 min/day at >3 METs (n = 188) | | | |  | ≥15 min/day at >3 METs (n = 150) | | | | |
| --- | --- | --- | --- | --- | --- | --- | --- | --- | --- | --- |
|  | Detection rate  (%) | Mean ± SD  (log_10_ cells/g feces) | | |  | Detection rate  (%) | Mean ± SD  (log_10_ cells/g feces) | | | |
| Obligate anaerobes |  |  |  |  |  |  | |  |  |  |
| *Clostridium coccoides* group | 100 | 9.8 | ± | 0.5 |  | 100 | | 9.8 | ± | 0.5 |
| *Clostridium leptum* subgroup | 100 | 10.0 | ± | 0.7 |  | 100 | | 9.9 | ± | 0.6 |
| *Bacteroides fragilis* group | 99 | 9.5 | ± | 0.7 |  | 99 | | 9.4 | ± | 0.8 |
| *Atopobium* cluster | 100 | 9.1 | ± | 0.7 |  | 100 | | 9.0 | ± | 0.7 |
| Genus *Bifidobacterium* | 100 | 9.3 | ± | 0.9 |  | 99 | | 9.4 | ± | 1.0 |
| Genus *Prevotella* | 66 | 6.8 | ± | 1.9 |  | 67 | | 6.8 | ± | 1.9 |
| *Clostridium perfringens* | 51 | 4.3 | ± | 1.5 |  | 49 | | 4.2 | ± | 1.4 |
| *Clostridium difficile* | 5 | 3.2 | ± | 0.5 |  | 2 | | 3.1 | ± | 0.4 |
| Facultative anaerobes |  |  |  |  |  |  | |  |  |  |
| Genus *Streptococcus* | 97 | 8.8 | ± | 1.1 |  | 98 | | 8.7 | ± | 1.1 |
| Family Enterobacteriaceae | 96 | 7.4 | ± | 1.1 |  | 95 | | 7.3 | ± | 1.1 |
| Genus *Enterococcus* | 85 | 6.4 | ± | 1.6 |  | 79 | | 6.1 | ± | 1.6 |
| Genus *Staphylococcus* | 57 | 4.6 | ± | 0.7 |  | 52 | | 4.5 | ± | 0.7 |
| Total *Lactobacillus* | 99 | 7.3 | ± | 1.2 |  | 99 | | 7.1 | ± | 1.1 |
| *Lactobacillus casei* subgroup | 68 | 5.6 | ± | 1.5 |  | 61 | | 5.5 | ± | 1.5 |
| *Lactobacillus gasseri* subgroup | 91 | 6.1 | ± | 1.6 |  | 94 | | 6.1 | ± | 1.4 |
| *Lactobacillus plantarum* subgroup | 78 | 4.6 | ± | 1.3 |  | 68 | | 4.4 | ± | 1.3 |
| *Lactobacillus reuteri* subgroup | 80 | 5.1 | ± | 1.5 |  | 75 | | 4.8 | ± | 1.5 |
| *Lactobacillus ruminis* subgroup | 41 | 5.4 | ± | 1.5 |  | 37 | | 5.1 | ± | 1.3 |
| *Lactobacillus sakei* subgroup | 39 | 4.0 | ± | 1.4 |  | 35 | | 3.8 | ± | 1.2 |
| *Lactobacillus fermentum* | 49 | 5.8 | ± | 1.0 |  | 34 | | 5.5 | ± | 0.9 |
| *Lactobacillus brevis* | 32 | 3.5 | ± | 1.0 |  | 31 | | 3.5 | ± | 0.9 |
| Aerobes |  |  |  |  |  |  | |  |  |  |
| Genus *Pseudomonas* | 13 | 4.2 | ± | 0.5 |  | 11 | | 4.2 | ± | 0.3 |

SD, standard deviation.

Independent differences in each bacterial count between groups were assessed by analyses of covariance, after adjusting data for age, sex, body mass index, smoking status and alcohol intake.

Data on step count are not shown, mainly because of their similarities to those on the duration of exercise >3 METs.

**Supplementary Table 7.** Relative abundances of the fecal bacteria families (limited to a share ≥0.1%) in subjects consuming fermented milk products containing *Lactobacillus casei* strain Shirota (LcS) 0-2, 3-5 or 6-7 days per week

|  | 0-2 days/week (n = 202) | | |  | 3-5 days/week (n = 53) | | |  | 6-7 days/week (n = 78) | | |
| --- | --- | --- | --- | --- | --- | --- | --- | --- | --- | --- | --- |
|  | Mean ± SD (%) | | |  | Mean ± SD (%) | | |  | Mean ± SD (%) | | |
| Phylum Firmicutes |  |  |  |  |  |  |  |  |  |  |  |
| Lachnospiraceae | 25.0 | ± | 10.0 |  | 24.2 | ± | 12.0 |  | 22.9 | ± | 8.3 |
| Ruminococcaceae | 15.5 | ± | 8.3 |  | 13.8 | ± | 7.8 |  | 15.1 | ± | 7.0 |
| Streptococcaceae | 2.5 | ± | 4.5 |  | 3.0 | ± | 4.5 |  | 2.9 | ± | 4.5 |
| Veillonellaceae | 2.1 | ± | 3.1 |  | 2.3 | ± | 2.5 |  | 1.6 | ± | 1.9 |
| Clostridiaceae | 1.8 | ± | 2.3 |  | 1.8 | ± | 4.1 |  | 1.5 | ± | 1.6 |
| o_Clostridiales_unclassified | 1.6 | ± | 1.9 |  | 1.2 | ± | 1.4 |  | 1.8 | ± | 1.9 |
| Lactobacillaceae | 0.84 | ± | 2.62 |  | 3.7 | ± | 9.8 |  | 1.4 | ± | 4.7 |
| Erysipelotrichaceae | 1.4 | ± | 1.7 |  | 1.2 | ± | 1.5 |  | 1.5 | ± | 1.8 |
| Enterococcaceae | 0.56 | ± | 5.80 |  | 0.07 | ± | 0.32 |  | 0.06 | ± | 0.24 |
| o_Clostridiales_Other | 0.31 | ± | 0.50 |  | 0.24 | ± | 0.40 |  | 0.43 | ± | 0.87 |
| Turicibacteraceae | 0.30 | ± | 0.68 |  | 0.24 | ± | 0.81 |  | 0.18 | ± | 0.44 |
| Christensenellaceae | 0.29 | ± | 0.90 |  | 0.14 | ± | 0.48 |  | 0.29 | ± | 0.97 |
| Bacillaceae | 0.22 | ± | 0.40 |  | 0.09 | ± | 0.13 † |  | 0.14 | ± | 0.28 |
| Mogibacteriaceae | 0.11 | ± | 0.19 |  | 0.10 | ± | 0.14 |  | 0.11 | ± | 0.11 |
| Phylum Bacteroidetes |  |  |  |  |  |  |  |  |  |  |  |
| Bacteroidaceae | 25.5 | ± | 15.1 |  | 23.7 | ± | 15.3 |  | 25.6 | ± | 14.9 |
| Prevotellaceae | 6.7 | ± | 14.4 |  | 9.1 | ± | 17.8 |  | 8.8 | ± | 16.6 |
| Porphyromonadaceae | 1.7 | ± | 2.0 |  | 2.0 | ± | 2.9 |  | 1.9 | ± | 1.8 |
| Rikenellaceae | 1.3 | ± | 1.9 |  | 1.0 | ± | 1.2 |  | 1.3 | ± | 1.4 |
| Paraprevotellaceae | 0.59 | ± | 1.64 |  | 0.69 | ± | 1.88 |  | 0.79 | ± | 1.75 |
| S24-7 | 0.39 | ± | 1.37 |  | 0.31 | ± | 0.75 |  | 0.36 | ± | 1.23 |
| Barnesiellaceae | 0.29 | ± | 0.48 |  | 0.32 | ± | 0.55 |  | 0.36 | ± | 0.58 |
| Odoribacteraceae | 0.21 | ± | 0.24 |  | 0.20 | ± | 0.21 |  | 0.28 | ± | 0.27 |
| Phylum Actinobacteria |  |  |  |  |  |  |  |  |  |  |  |
| Bifidobacteriaceae | 7.1 | ± | 10.1 |  | 6.7 | ± | 8.7 |  | 5.8 | ± | 7.7 |
| Coriobacteriaceae | 0.73 | ± | 0.79 |  | 0.69 | ± | 0.56 |  | 0.74 | ± | 0.70 |
| Phylum Proteobacteria |  |  |  |  |  |  |  |  |  |  |  |
| Enterobacteriaceae | 1.2 | ± | 3.1 |  | 1.7 | ± | 3.5 |  | 2.2 | ± | 5.1 |
| Alcaligenaceae | 0.70 | ± | 0.64 |  | 0.76 | ± | 0.61 |  | 0.74 | ± | 0.66 |
| Pasteurellaceae | 0.12 | ± | 0.37 |  | 0.11 | ± | 0.21 |  | 0.10 | ± | 0.23 |
| Phylum Fusobacteria |  |  |  |  |  |  |  |  |  |  |  |
| Fusobacteriaceae | 0.31 | ± | 1.30 |  | 0.12 | ± | 0.47 |  | 0.15 | ± | 0.52 |
| Phylum Verrucomicrobia |  |  |  |  |  |  |  |  |  |  |  |
| Verrucomicrobiaceae | 0.20 | ± | 0.53 |  | 0.14 | ± | 0.36 |  | 0.30 | ± | 0.98 |

SD, standard deviation.

Linear trends for three-group differences in each bacterial abundance were assessed by analyses of covariance, after adjusting data for age, sex, body mass index, smoking status and alcohol intake; if the trends were statistically significant, *post hoc* Tukey’s tests assessed two-group differences in each bacterial abundance.

† *P* < 0.05 versus 0-2 days/week.

Data on overall fermented milk products are not shown, mainly because of their similarities to those on LcS-containing products.

**Supplementary Table 8.** Relative abundances of the fecal bacteria families (limited to a share ≥0.1%) in subjects taking exercise demanding an energy expenditure >3 metabolic equivalents (METs) for <15 or ≥15 min per day

|  | <15 min/day at >3 METs (n = 184) | | |  | ≥15 min/day at >3 METs (n = 149) | | |
| --- | --- | --- | --- | --- | --- | --- | --- |
|  | Mean ± SD (%) | | |  | Mean ± SD (%) | | |
| Phylum Firmicutes |  |  |  |  |  |  |  |
| Lachnospiraceae | 24.6 | ± | 10.2 |  | 24.1 | ± | 9.8 |
| Ruminococcaceae | 15.0 | ± | 7.5 |  | 15.3 | ± | 8.4 |
| Streptococcaceae | 3.1 | ± | 4.8 |  | 2.2 | ± | 4.1 |
| Veillonellaceae | 1.8 | ± | 2.4 |  | 2.2 | ± | 3.2 |
| Clostridiaceae | 1.7 | ± | 2.6 |  | 1.8 | ± | 2.4 |
| o_Clostridiales_unclassified | 1.5 | ± | 1.5 |  | 1.7 | ± | 2.1 |
| Lactobacillaceae | 1.7 | ± | 6.2 |  | 1.1 | ± | 3.2 |
| Erysipelotrichaceae | 1.4 | ± | 1.7 |  | 1.4 | ± | 1.7 |
| Enterococcaceae | 0.10 | ± | 0.30 |  | 0.69 | ± | 6.75 |
| o_Clostridiales_Other | 0.36 | ± | 0.67 |  | 0.29 | ± | 0.49 |
| Turicibacteraceae | 0.26 | ± | 0.73 |  | 0.27 | ± | 0.55 |
| Christensenellaceae | 0.23 | ± | 0.75 |  | 0.31 | ± | 0.99 |
| Bacillaceae | 0.14 | ± | 0.33 |  | 0.22 | ± | 0.38 * |
| Mogibacteriaceae | 0.12 | ± | 0.20 |  | 0.10 | ± | 0.12 |
| Phylum Bacteroidetes |  |  |  |  |  |  |  |
| Bacteroidaceae | 25.6 | ± | 14.4 |  | 24.8 | ± | 15.8 |
| Prevotellaceae | 7.3 | ± | 15.0 |  | 8.0 | ± | 16.1 |
| Porphyromonadaceae | 1.8 | ± | 2.0 |  | 1.7 | ± | 2.2 |
| Rikenellaceae | 1.3 | ± | 1.5 |  | 1.2 | ± | 1.9 |
| Paraprevotellaceae | 0.69 | ± | 1.76 |  | 0.61 | ± | 1.64 |
| S24-7 | 0.33 | ± | 1.16 |  | 0.43 | ± | 1.37 |
| Barnesiellaceae | 0.32 | ± | 0.51 |  | 0.29 | ± | 0.52 |
| Odoribacteraceae | 0.24 | ± | 0.25 |  | 0.21 | ± | 0.24 |
| Phylum Actinobacteria |  |  |  |  |  |  |  |
| Bifidobacteriaceae | 6.2 | ± | 9.2 |  | 7.5 | ± | 9.5 |
| Coriobacteriaceae | 0.69 | ± | 0.63 |  | 0.78 | ± | 0.85 |
| Phylum Proteobacteria |  |  |  |  |  |  |  |
| Enterobacteriaceae | 1.7 | ± | 4.4 |  | 1.3 | ± | 2.7 |
| Alcaligenaceae | 0.69 | ± | 0.64 |  | 0.76 | ± | 0.64 |
| Pasteurellaceae | 0.13 | ± | 0.37 |  | 0.09 | ± | 0.24 |
| Phylum Fusobacteria |  |  |  |  |  |  |  |
| Fusobacteriaceae | 0.30 | ± | 1.32 |  | 0.16 | ± | 0.61 * |
| Phylum Verrucomicrobia |  |  |  |  |  |  |  |
| Verrucomicrobiaceae | 0.20 | ± | 0.56 |  | 0.23 | ± | 0.74 |

SD, standard deviation.

Independent differences in each bacterial abundance between groups were assessed by analyses of covariance, after adjusting data for age, sex, body mass index, smoking status and alcohol intake.

* *P* < 0.05 versus <15 min/day at >3 METs.

Data on step count are not shown, mainly because of their similarities to those on the duration of exercise >3 METs.

**References**

Aoyagi, Y., and Shephard, R. J. (2009). Steps per day: the road to senior health? *Sports Med*. 39, 423-438. doi: 10.2165/00007256-200939060-00001

Aoyagi, Y., and Shephard, R. J. (2010). Habitual physical activity and health in the elderly: the Nakanojo Study. *Geriatr. Gerontol. Int*. 10, S236–S243. doi: 10.1111/j.1447-0594.2010.00589.x

Aoyagi, Y., and Shephard, R. J. (2011). A model to estimate the potential for a physical activity-induced reduction in healthcare costs for the elderly, based on pedometer/accelerometer data from the Nakanojo Study. *Sports Med*. 41, 695-708. doi: 10.2165/11590530-000000000-00000

Aoyagi, Y., and Shephard, R. J. (2013). Sex differences in relationships between habitual physical activity and health in the elderly: practical implications for epidemiologists based on pedometer/accelerometer data from the Nakanojo Study. *Arch. Gerontol. Geriatr*. 56, 327-338. doi: 10.1016/j.archger.2012.11.006

Kato-Kataoka, A., Nishida, K., Takada, M., Kawai, M., Kikuchi-Hayakawa, H., Suda, K., et al. (2016). Fermented milk containing *Lactobacillus casei* strain Shirota preserves the diversity of the gut microbiota and relieves abdominal dysfunction in healthy medical students exposed to academic stress. *Appl. Environ. Microbiol*. 82, 3649-3658. doi: 10.1128/AEM.04134-15

Kikuchi, E., Miyamoto, Y., Narushima, S., and Itoh, K. (2002). Design of species-specific primers to identify 13 species of *Clostridium* harbored in human intestinal tracts. *Microbiol. Immunol*. 46, 353-358. doi: 10.1111/j.1348-0421.2002.tb02706.x

Matsuda, K., Tsuji, H., Asahara, T., Kado, Y., and Nomoto, K. (2007). Sensitive quantitative detection of commensal bacteria by rRNA-targeted reverse transcription-PCR. *Appl. Environ. Microbiol*. 73, 32–39. doi: 10.1128/AEM.01224-06

Matsuda, K., Tsuji, H., Asahara, T., Matsumoto, K., Takada, T., and Nomoto, K. (2009). Establishment of an analytical system for the human fecal microbiota, based on reverse transcription-quantitative PCR targeting of multicopy rRNA molecules. *Appl. Environ. Microbiol*. 75, 1961–1969. doi: 10.1128/AEM.01843-08

Matsuda, K., Tsuji, H., Asahara, T., Takahashi, T., Kubota, H., Nagata, S., et al. (2012). Sensitive quantification of *Clostridium difficile* cells by reverse transcription-quantitative PCR targeting rRNA molecules. *Appl. Environ. Microbiol*. 78, 5111-5118. doi: 10.1128/AEM.07990-11

Matsuki, T. (2007). Development of quantitative PCR detection method with 16S rRNA gene-targeted genus- and species-specific primers for the analysis of human intestinal microflora and its application. *Jpn. J. Bacteriol*. 62, 255-261. (in Japanese). doi: 10.3412/jsb.62.255

Matsuki, T., Watanabe, K., Fujimoto, J., Takada, T., and Tanaka, R. (2004). Use of 16S rRNA gene-targeted group-specific primers for real-time PCR analysis of predominant bacteria in human feces. *Appl. Environ. Microbiol*. 70, 7220-7228. doi: 10.1128/AEM.70.12.7220-7228.2004

Sakaguchi, S., Saito, M., Tsuji, H., Asahara, T., Takata, O., Fujimura, J., et al. (2010). Bacterial rRNA-targeted reverse transcription-PCR used to identify pathogens responsible for fever with neutropenia. *J. Clin. Microbiol*. 48, 1624-1628. doi: 10.1128/JCM.01724-09

Watanabe, K. (1998). Primers for *Lactobacillus*. Japan Patent Application No. 1998-260041; Publication No. 1999-151097. Chiyoda, Tokyo: Japan Patent Office.
